# The prevalence and association of chronic kidney disease and diabetes in liver cirrhosis using different estimated glomerular filtration rate equation

**DOI:** 10.18632/oncotarget.23368

**Published:** 2017-12-18

**Authors:** Cheng-Yi Chen, Cheng-Jui Lin, Chih-Sheng Lin, Fang-Ju Sun, Chi-Feng Pan, Han-Hsiang Chen, Chih-Jen Wu

**Affiliations:** ^1^ Division of Nephrology, Department of Internal Medicine, Mackay Memorial Hospital, Hsinchu, Taiwan; ^2^ MacKay Junior College of Medicine, Nursing and Management, Taipei, Taiwan; ^3^ Department of Biological Science and Technology, National Chiao Tung University, Hsinchu, Taiwan; ^4^ Division of Nephrology, Department of Internal Medicine, Mackay Memorial Hospital, Taipei, Taiwan; ^5^ Medicine, College of Medicine, Taipei Medical University, Taipei, Taiwan; ^6^ Department of Medical Research, MacKay Memorial Hospital, Taipei, Taiwan; ^7^ Graduate Institute of Medical Sciences and Department of Pharmacology, School of Medicine, College of Medicine, Taipei Medical University, Taipei, Taiwan; ^8^ Department of Medical Research, China Medical University Hospital, China Medical University, Taichung, Taiwan

**Keywords:** liver cirrhosis, chronic kidney disease, diabetes mellitus, estimated glomerular filtration rate, MELD score

## Abstract

**Background:**

Chronic kidney disease (CKD) in cirrhosis is one of the dreaded complications associated with a steep rise in mortality and morbidity, including diabetes. There are limited data on the prevalence of CKD and the association with diabetes in outpatients with cirrhosis.

**Methodology:**

This is a cross-sectional study of 7,440 adult liver cirrhosis patients enrolled from August 2001 to April 2010 in a medical center. Case control matching by age and sex with 1,967 pairs, and conditional logistic regression for odds of diabetes was analyzed using adjusted model.

**Results:**

CKD was present in 46.0%, 45.7% and 45.6% of the study population using the MDRD-6, CKD-EPI and MDRD-4 estimated glomerular filtration rate (eGFR) equations, respectively. Using a conditional logistic regression model after adjusting for other risk factors, odds for diabetes increased significantly compared with non-CKD in CKD stage 3 to 5 (stage 3~5) based on MDRD-6–adjusted model, ORs were: stage 3~5, 2.34 (95% CI, 1.78-3.01); MDRD-4–adjusted model, ORs were: stage 3~5, 8.51 (95% CI, 5.63-11.4); CKD-EPI–adjusted model, ORs were: stage 3~5, 8.61 (95% CI, 5.13-13.9).

**Conclusion:**

In cirrhosis patients, prevalence of diabetes was higher in patients with advanced stage of CKD. For patients with cirrhosis, patients with CKD stages 3~5 defined by MDRD-4, MDRD-6, and CKD-EPI eGFR equations had increased risk for diabetes. More severe cirrhosis, indicated by the Child-Turcott-Pugh classification was also accompanied by an increased risk for diabetes.

## INTRODUCTION

Liver cirrhosis often requires liver transplantation for treatment, as the main cause of mortality along with chronic liver disease. The low survival rate of decompensated cirrhotic people has driven research for good prognostic markers [1]. In patients with liver cirrhosis, renal function has important prognostic impact along with hepatic function [2]. Furthermore, Advanced CKD in liver cirrhosis associated with proceeding to end stage renal disease (ESRD) even after liver transplantation [3]. The most virulent sequence of cirrhosis is hepatorenal syndrome (HRS), identified by an acute or subacute progression in kidney function [4].

CKD is a notorious health issue in Taiwan with high prevalence (11.93%), yet low awareness (3.54%) of the condition [5]. However, only few researches had investigated CKD prevalence in Taiwan cirrhosis patients, and more research needs to be conducted. In a general population, the CKD Epidemiology Collaboration (CKD-EPI) equation [6] is a better estimate of measured glomerular filtration rate (GFR) at higher GFR levels than the Modification of Diet in Renal Disease (MDRD) Study equation. However, the GFR calculated by the 6-variable MDRD equation may be closer to the true GFR than that calculated by the CKD-EPI equation in liver cirrhosis [7]. Prevalence of CKD within cirrhosis of one Taiwan medical center was analyzed via three different equations of estimated GFR (eGFR).

Gathering evidence points that CKD, especially ESRD, including that after kidney transplantation and hemodialysis therapy initiation, is associated with newly diagnosed diabetes, [8–10]. However, no data is currently available on the prevalence of CKD and diabetes in outpatients with cirrhosis.

This study aimed to provide a comprehensive analysis of CKD prevalence and diabetes in a large cohort of outpatients with cirrhosis. The relationship between CKD and diabetes mellitus(DM) in cirrhosis patients is also discussed.

## RESULTS

### Baseline characteristics of the cirrhotic cohort

For the included 7,440 eligible enrolled patients, demographic, laboratory, and clinical data were collected between August 2000 and April, 2010. Mean age was 62.8±14.3 years (Table 1). Of the total cohort, 67.5% was male. Among the etiology of cirrhosis, viral hepatitis was 42.3%, alcoholic was 37.6%, alcoholic with viral hepatitis was 12.3% and cryptogenic was 7.8%. The severities of liver disease according to the CTP classification were as follows: Child class A was 34.2%, Child B was 57.7% and Child C was 8.1%. The median (interquarter range) of MELD score was 20 (19) and 16 (16) in DM and non-DM respectively. Three estimated glomerular filtration equation were compared: highest one CKD-EPI median (interquarter range): 65.0 (62.9), 78.0 (53.6) and lowest one MDRD-6: 51.0 (52.2), 60.5 (46.9) mL/min/1.73 m^2^ in DM and non-DM respectively.

**Table 1:** Baseline characteristics and parameter of the pooled and matched cohort

	Pooled cohort			Matched cohort		
Characteristics	DM (n=1,967)	Non-DM (n=5,473)	p value	DM (n=1,967)	Non-DM (n=1,967)	p value
Age (years)	66 (19)	64 (16)	<0.001	66 (19)	66 (19)	0.342
Male N (%)	1,216 (61.8)	3,809 (69.6)		1,216 (61.8)	1,216 (61.8)	
Female N (%)	751 (38.2)	1,664 (30.4)		751 (38.2)	751 (38.2)	
HCV N (%)	470(23.9)	1110 (20.3)		470(23.9)	437(22.2)	
Proteinuria N (%)	1337(68)	2298(42)		1337(68)	768(39)	
Hypertension	1624(82.6)	3683(67.3)		1624(82.6)	1368(69.5)	
Ammonia (μg/dL)	56(13)	43(11)	<0.001	56(13)	48(12)	<0.001
BMI (Kg/m^2^)	27.6(3)	23.5(3)	<0.001	27.4(3)	23.5(3)	<0.001
FPG (mg/dL)	130 (82)	103 (58)	<0.001	130 (82)	105 (66)	<0.001
Hb (g/dL)	10.7 (4)	11.2 (4)	<0.001	10.4 (3.5)	11.8 (3.7)	<0.001
Platelet (10^3^/uL)	114 (100)	121 (112)	0.005	116 (104)	135 (97)	<0.001
AST (IU/L)	54 (70)	51 (62)	0.013	49 (57)	42 (39)	<0.001
ALT (IU/L)	33 (36)	33 (34)	0.929	32 (32)	31 (29)	0.566
Albumin (g/dL)	54 (70)	51 (62)	<0.001	2.9 (1.0)	3.3 (1.0)	<0.001
Total bilirubin (mg/dL)	3 (1.2)	3.1 (1.2)	<0.001	2.5 (2)	1.3 (1)	<0.001
Alk-Phosphate (IU/L)	98 (72)	94 (70)	0.018	100 (71)	87 (57)	<0.001
Uric Acid (mg/dL)	6.2 (3.1)	6.0 (2.9)	0.001	6.3 (3.3)	5.6 (2.6)	<0.001
BUN (mg/dL)	18 (30)	15 (17)	<0.001	24 (36)	12 (8)	<0.001
Cr (mg/dL)	1.1 (1.2)	1.0 (0.6)	<0.001	1.3 (1.5)	0.8 (0.3)	<0.001
Cholesterol (mg/dL)	143 (70)	143 (69)	0.745	146 (69)	146 (63)	0.092
TG (mg/dL)	88 (62)	88 (64)	0.631	95 (73)	91 (72)	0.823
Sodium (mg/dL)	137 (6)	138 (6)	0.168	137 (6)	138 (5)	0.039
Potassium (mg/dL)	4.0 (1.0)	4.0 (0.9)	0.144	4.1 (0.9)	4.0 (0.7)	<0.001
Chloride (mg/dL)	105 (9)	105 (8)	0.733	105 (9)	106 (8)	0.019
INR	1.25 (0.51)	1.22 (0.45)	<0.001	1.24 (0.51)	1.14 (0.27)	<0.001
MELD score	20 (19)	16 (16)	<0.001	20 (19)	9 (6)	<0.001
CTP score	7 (2)	7 (2)	<0.001	7 (2)	7 (1)	<0.001
MDRD-4 (ml/min)	62.4 (58.4)	73.2 (54.5)	<0.001	62.4 (58.4)	85.5 (27)	<0.001
MDRD-6 (ml/min)	51.0 (52.2)	60.5 (46.9)	<0.001	51 (52.2)	70.5 (23.4)	<0.001
CKD-EPI (ml/min)	65.0 (62.9)	78.0 (53.6)	<0.001	65 (62.9)	88.2 (20.8)	<0.001

Categorical data were presented as number(%), continuous data were expressed as median and interquartile range. The Mann-Whitney test and chi-square test were used.

Abbreviation: FPG, fasting plasma glucose; Hb, hemoglobin; AST, aspartate amino transferase; ALT, alanine amino transferase; BUN, blood urea nitrogen; Cr, creatinine; TG, triglyceride.

INR, international normalized ratio; CTP, Child-Turcott-Pugh; MELD, Model for End-Stage Liver Disease; INR, international normalized ratio; MDRD, Modification of Diet in Renal Disease; CKD-EPI, Chronic Kidney Disease Epidemiology Collaboration.

Of 7,440 pooled cohort, 1,967 (35.9%) had diabetes (Table 1). In matched cohort, Median and interquartile range of fasting blood glucose level was 103 (58) mg/dL for nondiabetic and 105 (66) mg/dL for diabetic participants (p<0.001). In diabetic group, there are lower hemoglobin, platelet count, albumin, lower estimated glomerular rate in three different equations. Higher AST, total bilirubin, alkaline phosphatase, uric acid, blood urea nitrogen (BUN), creatinine, potassium, international normalized ratio (INR) and MELD score and CTP score was significantly noted in diabetes matched group.

### Prevalence of diabetics in different CKD stage compared by three eGFR equations

**Figure 1:**
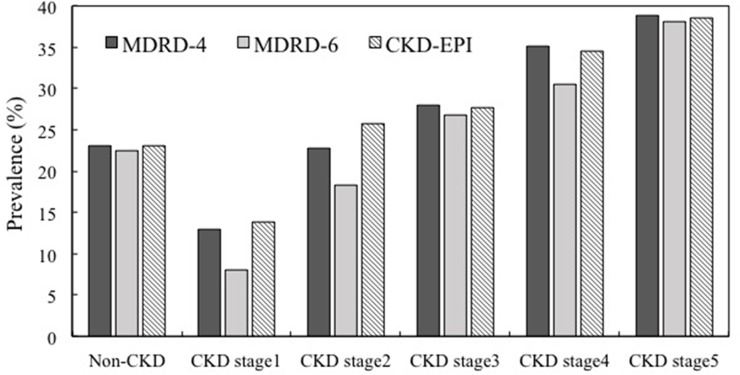
Prevalence of different CKD stage in MDRD-4, MRDR-6, and CKD-EPI Estimating GFR (eGFR) Equations

The prevalence of diabetics in non-CKD and different CKD stages was listed in Table 2 and compared with three different eGFR equations. For people known as having CKD, diabetes prevalence distinctly increased (34.9%, MDRD-6; 30.6%, MDRD-4; 30.5%, CKD-EPI equation). In CKD patients, diabetes had higher prevalence rate using MDRD-6 compared other two equations which had similar results. In Non-CKD patients, diabetes had lower prevalence in MRDR6 compared other two equations (22.5% versus 23%). In CKD cohort, the trend of diabetes prevalence was increased from CKD stage 1 to stage 5. The prevalence was highest in CKD stage 1 and 2 using CKD-EPI and highest in CKD stage 3~5 when using MDRD-4 equation (Figure 1).

**Table 2:** Prevalence of diabetes in different CKD stage in MDRD-4, MDRD-6, and CKD-EPI estimating GFR equations

	Non- CKD	All CKD	CKD stage 1	CKD stage 2	CKD stage 3	CKD stage 4	CKD stage 5
			MDRD-4 equation	p<0.001			
			MDRD-6 equation	p<0.001			
			CKD-EPI equation	p<0.001			
Total N (%)	4,044	3,396 (45.6%)	70	400	1,580	709	637
Diabetics	929	1,038	9	91	442	249	247
Diabetics (%)	23.0%	30.6%	12.9%	22.8%	28.0%	35.1%	38.8%
Total	4,004	3,421 (46.0%)	37	109	2,075	855	928
Diabetics	770	1,194	3	20	556	261	354
Diabetics (%)	22.5%	34.9%	8.1%	18.3%	26.8%	30.5%	38.1%
Total	4,040	3,400 (45.7%)	94	489	1,461	682	674
Diabetics	928	1,039	13	126	405	235	260
Diabetics (%)	23.0%	30.5%	13.8%	25.8%	27.7%	34.5%	38.6%

Statistical comparison was performed with One-way analysis of variance

Abbreviations: CKD, chronic kidney disease; CKD-EPI, Chronic Kidney Disease Epidemiology Collaboration; MDRD, Modification of Diet in Renal Disease; MELD, Model for End-Stage Liver Disease; CTP, Child-Turcott-Pugh.

### Prevalence of diabetics in different group MELD score and CTP classification

**Figure 2:**
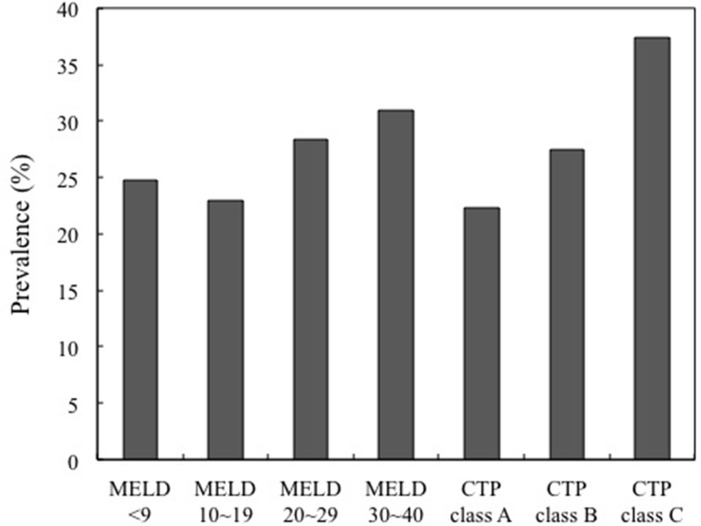
Prevalence of diabetics in different group MELD score and CTP classification

Prevalence of diabetes in different group MELD and CTP classification were calculated. MELD group 4 had highest prevalence rate of 31% and group 2 had lowest prevalence rate 23%. The prevalence was increased in trend from CTP class A to C (A:22.3%, B:27.4%, and C:37.4%) (Figure 2). The prevalence of diabetes increased from mild (CTP class B) to severe (CTP class C) in the cirrhosis group, as shown in Table 3. A higher prevalence rate of advanced CKD with increased severity of MELD scores was also observed (Table 4).

**Table 3:** Prevalence of diabetics in different group MELD score and CTP classification

**MELD**	**Group 1**	**Group 2**	**Group 3**	**Group 4**	**p for trend**
Total	2,015	1,989	1,986	1,450	p<0.001
Diabetics	497	458	562	450	
**CTP class**	**Class A**	**Class B**	**Class C**		
Diabetics (%)	24.7%	23.0%	28.3%	31.0%	
Total	2,547	4,291	602		p<0.001
Diabetics	567	1,175	225		
Diabetics (%)	22.3%	27.4%	37.4%		

Statistical comparison was performed with One-way analysis of variance.

Abbreviations: MELD, Model for End-Stage Liver Disease; CTP, Child-Turcott-Pugh.

**Table 4:** Prevalence of CKD Stage with different group divided as MELD score (Group 1: MELD <9; Group 2: MELD 10~19; Group 3: MELD 20~29; Group 4: MELD 30~40)

Characteristics	Group 1 n=2,015	Group 2 n=1,989	Group 3 n=1,986	Group 4 n=1,450
MELD score	7.6±1.1	13.3±3.0	23.3±2.7	38.6±6.0
MDRD-4 equation ( percentage % and number)				
CKD stage 1	1.1% (23)	1.2% (23)	0.6% (12)	0.8% (12)
CKD stage 2	3.8% (77)	5.3% (106)	8.1% (160)	3.9% (57)
CKD stage 3	5.7% (114)	9.6% (191)	41.6% (826)	31.0% (449)
CKD stage 4	0.6% (12)	1.5% (30)	12.4% (247)	29.0% (420)
CKD stage 5	0.4% (8)	1.0% (20)	11.0% (218)	27.0% (391)
MDRD-6 equation				
CKD stage 1	0.4% (9)	0.6% (11)	0.4% (7)	0.7% (10)
CKD stage 2	1.8% (37)	1.9% (37)	1.4% (27)	0.6% (8)
CKD stage 3	18.7% (376)	21.8% (433)	46.5% (923)	23.9% (343)
CKD stage 4	0.6% (13)	2.3% (46)	19.1% (378)	29.1% (418)
CKD stage 5	0.6% (13)	1.5% (29)	15.7% (311)	40.0% (575)
CKD-EPI equation				
CKD stage 1	1.4% (29)	1.6% (32)	0.9% (17)	1.1% (16)
CKD stage 2	4.7% (94)	6.4% (127)	10.2% (203)	4.5% (65)
CKD stage 3	4.6% (93)	8.2% (164)	38.9% (773)	29.7% (431)
CKD stage 4	0.5% (10)	1.4% (27)	12.2% (243)	27.7% (402)
CKD stage 5	0.4% (9)	1.0% (20)	11.5% (228)	28.8% (417)

Abbreviations: CKD, chronic kidney disease; CKD-EPI, Chronic Kidney Disease Epidemiology Collaboration; MDRD, Modification of Diet in Renal Disease; MELD, Model for End-Stage Liver Disease; CTP, Child-Turcott-Pugh.

### Prevalence of different CKD stage in different MELD score compared by three different eGFR equations

The severity of cirrhosis was assessed using MELD score and MELD score was calculated for every participant by

[MELD score = 9.6 × loge (creatinine mg/dL) + 3.8 × loge (bilirubin mg/dL) + 11.20 × loge (INR) + 6.4]

Participants were distributed into five groups accordingly.

Group I included of 5 participants with MELD score <9.

Group II included of 40 participants with MELD score = 10–19.

Group III included of 31 participants with MELD score = 20–29.

Group IV consisted of 19 patients with MELD score = 30–40.

Among the cirrhotic patient with CKD, CKD stage 3 had highest prevalence in all four groups. CKD prevalence increased in trend from group 1 to 4. Noticeably the prevalence rates of CKD stage 4 and 5 were increased in trend with the severity of cirrhosis in three different eGFR equations.

Using adjusted logistic regression model, age, female, CKD stage 4 and 5, obesity, proteinuria, HCV, hypertension, CVD and CTP class B and C had significantly higher odds for diabetes in three eGFR equations (Table 5).

**Table 5:** Logistic regression for diabetics in pooled cohorts

Characteristics	Adjusted model^a^	Adjusted model^b^	Adjusted model^c^
Equation	MDRD-4	MDRD-6	CKD-EPI
Non-CKD	1.00 (reference)	1.00 (reference)	1.00 (reference)
CKD stage 1	0.66 (0.30-1.45)	0.48 (0.13-1.75)	0.62 (0.30-1.27)
CKD stage 2	0.92 (0.68-1.24)	0.58 (0.30-1.05)	1.10 (0.90-1.41)
CKD stage 3	1.13 (0.92-1.39)	1.11 (0.92-1.31)	1.12 (0.93-1.33)
CKD stage 4	1.61 (1.25-2.11)	1.23 (1.03-1.44)	1.53 (1.16-2.01)
CKD stage 5	1.73 (1.31-2.26)	1.78 (1.44-2.12)	1.63 (1.24-2.15)
Age (per 10-yr increment)	1.11 (1.06-1.16)	1.11 (1.06-1.16)	1.11 (1.06-1.16)
Sex (reference:women)	0.85 (0.75-0.97)	0.85 (0.75-0.97)	0.85 (0.75-0.97)
Obesity^d^	1.74 (1.51-1.99)	1.74 (1.51-1.99)	1.74 (1.51-1.99)
Proteinuria	1.67(1.34-1.98)	1.67(1.34-1.98)	1.67(1.34-1.98)
HCV	2.03(1.78-2.28)	2.03(1.78-2.28)	2.03(1.78-2.28)
Hypertension	1.32(1.13-1.52)	1.32(1.13-1.52)	1.32(1.13-1.52)
Cardiovascular disease	1.29 (1.12-1.48)	1.29 (1.12-1.48)	1.29 (1.12-1.48)
Dyslipidemia^e^	1.06 (0.89-1.24)	1.06 (0.89-1.24)	1.06 (0.89-1.24)
Hemoglobin	1.02 (0.98-1.06)	1.02 (0.98-1.06)	1.02 (0.98-1.06)
Albumin	0.91 (0.80-1.03)	0.91 (0.80-1.03)	0.91 (0.80-1.03)
Uric acid	1.03(0.97-1.09)	1.03(0.97-1.09)	1.03(0.97-1.09)
Ammonia	1.21(0.96-1.48)	1.21(0.96-1.48)	1.21(0.96-1.48)
CTP class A	1.00 (reference)	1.00 (reference)	1.00 (reference)
CTP class B	1.23 (1.10-1.46)	1.23 (1.10-1.47)	1.23 (1.10-1.47)
CTP class C	1.45 (1.19-1.82)	1.48 (1.22-1.86)	1.47 (1.20-1.84)

Note: Values shown are odds ratio (95% confidence interval). Logistic regression, dependent variable diabetes/no diabetes; diabetes defined as self-reported, using medication, fasting glucose level >126 mg/dL, or nonfasting glucose level >200 mg/dL.

Abbreviations: OR, odds ratio; CI, confidence interval; CKD, chronic kidney disease; CKD-EPI, Chronic Kidney Disease Epidemiology Collaboration; MDRD, Modification of Diet in Renal Disease; MELD, Model for End-Stage Liver Disease; CTP, Child-Turcott-Pugh. Adjusted model for age, sex, Obesity, proteinuia, HCV, hypertension, CVD, dyslipidemia, hemoglobin, albumin, uric acid, ammonia and CTP class.

^a^MDRD-4 Study equation. ^b^MDRD-6 Study equation. ^c^CKD-EPI equation.^d^Body mass index >30 kg/m^2.^ ^e^Dyslipidemia defined as cholesterol level >200 mg/dL or triglyceride level >150 mg/dL.

In conditional logistic regression model, Using model 1 (MDRD-4), compared with the non-CKD participants, ORs for diabetes in CKD were: stage 1, 6.73 (95% CI, 1.27-47.4.); stage 2, 2.06 (95% CI, 1.26-3.35); stage 3~5, 8.51 (95% CI, 5.63-11.4). Using model 2 (MDRD-6), compared with the non-CKD participants, ORs for diabetes in CKD were: stage 1, 2.06 (95% CI, 0.24-16.2); stage 2, 1.31 (95% CI, 0.40-3.35); stage 3~5, 2.34 (95% CI, 1.78-3.01). Using model 3 (CKD-EPI), compared with the non-CKD participants, ORs for diabetes in CKD were: stage 1, 2.74 (95% CI, 0.67-9.2); stage 2, 2.56 (95% CI, 1.45-4.21); stage 3~5, 8.61(95% CI, 5.13-13.9) (Table 6).

**Table 6:** Conditional logistic regression for diabetics in matched cohorts

Characteristics	Adjusted model^a^	Adjusted model^b^	Adjusted model^c^
Equation	MDRD-4	MDRD-6	CKD-EPI
Non-CKD	1.00 (reference)	1.00 (reference)	1.00 (reference)
CKD stage 1	6.73 (1.27-47.4)	2.06 (0.24-16.2)	2.74 (0.67-9.2)
CKD stage 2	2.06 (1.26-3.35)	1.31 (0.45-3.35)	2.56 (1.45-4.21)
CKD stage 3~5	8.51 (5.63-11.4)	2.34 (1.78-3.01)	8.61 (5.13-13.9)
CTP class A	1.00 (reference)	1.00 (reference)	1.00 (reference)
CTP class B	1.46 (1.18-1.82)	1.44 (1.16-1.80)	1.47 (1.19-1.83)
CTP class C	2.33 (1.44-3.86)	2.64(1.63-4.22)	2.27 (1.35-3.78)

Abbreviations: OR, odds ratio; CI, confidence interval; CKD, chronic kidney disease; CKD-EPI, Chronic Kidney Disease Epidemiology Collaboration; MDRD, Modification of Diet in Renal Disease. CTP, Child-Turcott-Pugh.

Adjusted model for age, sex, obesity, proteinuia, HCV, hypertension, CVD, dyslipidemia, hemoglobin, albumin, uric acid, ammonia and CTP class.

^a^MDRD-4 Study equation. ^b^MDRD-6 Study equation. ^c^CKD-EPI equation.

## DISCUSSION

Using the MELD score, previous studies have shown that besides being a marker of liver function, serum creatinine(SCr) is useful in determining the prognosis of cirrhosis [11, 12]. Independent of hypo-perfusion and ischemia, cirrhotic popupation often have comorbidities that may cause CKD. In our analysis, eGFR was the lowest when calculated using the MDRD-6 equation, perhaps indicating a higher CKD prevalence rate. MDRD-6 equation is considered the best parameters in cirrhotic people, possibly because BUN and albumin levels are incorporates [13, 14]. Accumulating researches showed higher prevalence of DM in higher stage CKD [8, 9] and our data show a similar trend of increase in diabetes prevalence in cirrhosis patients as that seen in CKD patients, and across stages using the three different equations.

Cirrhotic patients are at risk for developing DM, mainly those with HCV [15] which in line with our result. Patients with cirrhosis and diabetes had poor outcome and higher risk for developing hepatocellular carcinoma than non-DM cirrhosis patients [16, 17]. The prevalence of diabetes increased from mild to severe in the cirrhosis group, as shown in Table 3. Chronic viral hepatitis and cirrhosis are identified to increased the risk of DM [18]. So-called “hepatogenous diabetes (HD)” is the mechanisms associated with insulin resistance and β-cell dysfunction that acquired with cirrhosis deterioration [19]. Perseghin et al. [20] found lessening insulin resistance and recovery HD in 67% of cirrhotic-diabetic patients after liver transplantation. Impaired removal of insulin by the injury liver and porto-systemic shunts make hyperinsulinemia in cirrhotic patient. It is exacerbated by increases in glucagon, growth hormone, insulin-like growth factor, free fatty acids, and cytokines [17, 21]. Liver cirrhosis characterized by a striking peripheral insulin resistance, and DM happened when the beta cells unable to recompense for the secretory performance [22–24]. Our data illustrated that increasing severity of cirrhosis significantly increased the risk of diabetes according to CTP classification (Tables 5 and 6). One study results showed that impairment of insulin secretion, but not of insulin sensitivity, associated with severity of cirrhosis, as evaluated by CTP score, which independently predicted β-cell dysfunction [25]. Clinical characteristics of HD differed from that of hereditary type 2 DM for less frequently related to retinopathy, cardiovascular and renal complications [26]. Prevention of HD is crucial in clinical practice for cirrhosis patient.

The MELD score incorporated creatinine, and we found a higher prevalence rate of advanced CKD with increased severity of MELD scores (Table 4). A retrospective study by Choi et al. [27]that investigated renal dysfunction, concluded that renal derangement in cirrhosis is not uncommon. Chronic viral hepatitis and cirrhosis frequently cause glomerular injury [28]. Besides, hypo-perfusion is a central mechanism in most patients with advanced cirrhosis and HRS also cause renal function deterioration [29]. Noticeably, the prevalence rate of higher stage CKD (stage 4 and 5) in the MDRD-6 equation was higher than that using the other equations (Table 4). Since the MDRD-6 equation was better than other equations in identifying cirrhosis patients with true GFR<30 mL/min/1.73 m^2^ [14], overestimating renal function using inappropriate eGFR equation was an important issue needed more attention.

Results indicate a higher OR of diabetes in CKD stage 4 and 5 parcipitants after multivariable adjustment.(Table 5). The results are essential since hyperglycemia in CKD people is related with a ominous process regarding morbidity and mortality [8, 30, 31]. Earlier detection of DM and strictly treat for CVD risk factors cant be ignored in reducing the CVD burden and preventing CKD deterioration [32].

We found increased risk of diabetes seen in CKD stage 3~5 using age- and sex-matched conditional logistic regression, adjusted for age, sex, Obesity, proteinuria, HCV, hypertension, CVD, dyslipidemia, hemoglobin, albumin, uric acid, ammonia and CTP class.(Table 6). Non-traditional risk factors for diabetes, including accumulated cytokines, oxidative stress; micro-albuminuria; insulin resistance; and endothelial malfunction were increased in higher stage CKD [33]. Traditional risk factors include aging, sex, ethnicity and obesity for diabetes were worsened after initiation of dialysis. Several studies in line with our finding that chronic inflammation is not uncommon in hemodialysis participants and it may tend to insulin resistance [34–36]. Besides, uremia related beta-cell virulence is also believed as a non-traditional risk factor for diabetes occurrence. However, two studies found controversial results that in lower eGFR or CKD, beta cell was suitably strengthening and risks of diabetes incidence were not significantly changed [37, 38].

The leading cause of mortality is hepatic failure among diabetic patients with cirrhosis [41, 42]. Bad glycemic management exist at cirrhosis diagnosis increased cirrhosis mortality and morbidity [43]. Our study suggests that prevented CKD progression and strictly cirrhosis treatment (albumin, ascites, hepatic encephalopathy, and coagulopathy correction) are important for managing cirrhosis, and perhaps more importantly, before diabetes development.

Our study was not without limitations. Using data from a single center, our analyses were cross-sectional and retrospective observational study. The relationship between the higher CKD stage and higher prevalence of diabetes is clear independent of eGFR equations, but the causal relationship cannot be determined by the findings of the study, because of the nature of a cross-sectional analysis. Analysis of data from larger multicenter patient clinics can discern observational biases and record errors that can arise within the current study. Yet, we use a detailed patient database permitted uniform evaluation and management for CKD and cirrhosis. Only patients who attended health evaluation and were aware of their condition were included, minimizing self-report limitations.

Furthermore, cirrhosis patients tend to have falsely low SCr levels due to decreases in hepatic creatinine synthesis and skeletal muscle mass. Factors that might increase the prevalence of DM in CKD participants include inflammatory and oxidative stress biomarkers, which were not measured and might therefore be confounding factors. Therefore, there may be some bias in underestimating the true prevalence and associated clinical manifestations of CKD may be underestimated.

A selection bias for patients whose data was in the pooled database could also have occurred. Studies on larger representative populations will overcome this limitation. Further investigations should take these factors into account, and a validation in translating such data into more robust longitudinal outcomes may also be warranted.

In conclusion, the present study suggests that CKD stages 3~5 defined by MDRD-4, MDRD-6 and CKD-EPI eGFR equations, are significantly and positively associated with diabetes. More severe cirrhosis in CTP classification also increased the risk for diabetes. Prospective cohort studies and clinical trials are required to further evaluate the causal relationship. New concepts on HD, the DM–HCV relationship, and clinical significance of DM in cirrhosis patients, also need to be confirmed with a large randomized control study.

## MATERIALS AND METHODS

### Study population and data collection

This is a retrospective cross section study that included consecutive, adult, cirrhotic patients from August 2001 to April 2010. The protocol was approved by the institutional review board of Mackay Memorial Hospital (IRB approval number: 10MMHIS172) and informed consent was waived. In addition to laboratory test features of hepatic dysfunction and the existence of major complications of liver cirrhosis, such as ascites, hepatic encephalopathy and gastroesophageal varices, were examined. The diagnosis of liver cirrhosis was either confirmed by liver biopsy or based on a combination of laboratory, endoscopic, and clinical features of portal hypertension together with compatible imaging findings (CT scan or ultrasound) or histological findings. We collected data as classified by the International Classification of Diseases, 9th revision (ICD-9) of “5712”, “5715”, “5716” from four branch of one medical center in Taiwan. For these 12,899 patients, patients with incomplete data with respect to renal function, age <18 years old were excluded; thus, a total of 7,440 patients was included in the study. The baseline characteristics documented on the subjects included age, gender, etiology of liver disease. Laboratory measures of liver function, renal function and electrolytes were obtained.

### Selection of cases and controls

**Figure 3:**
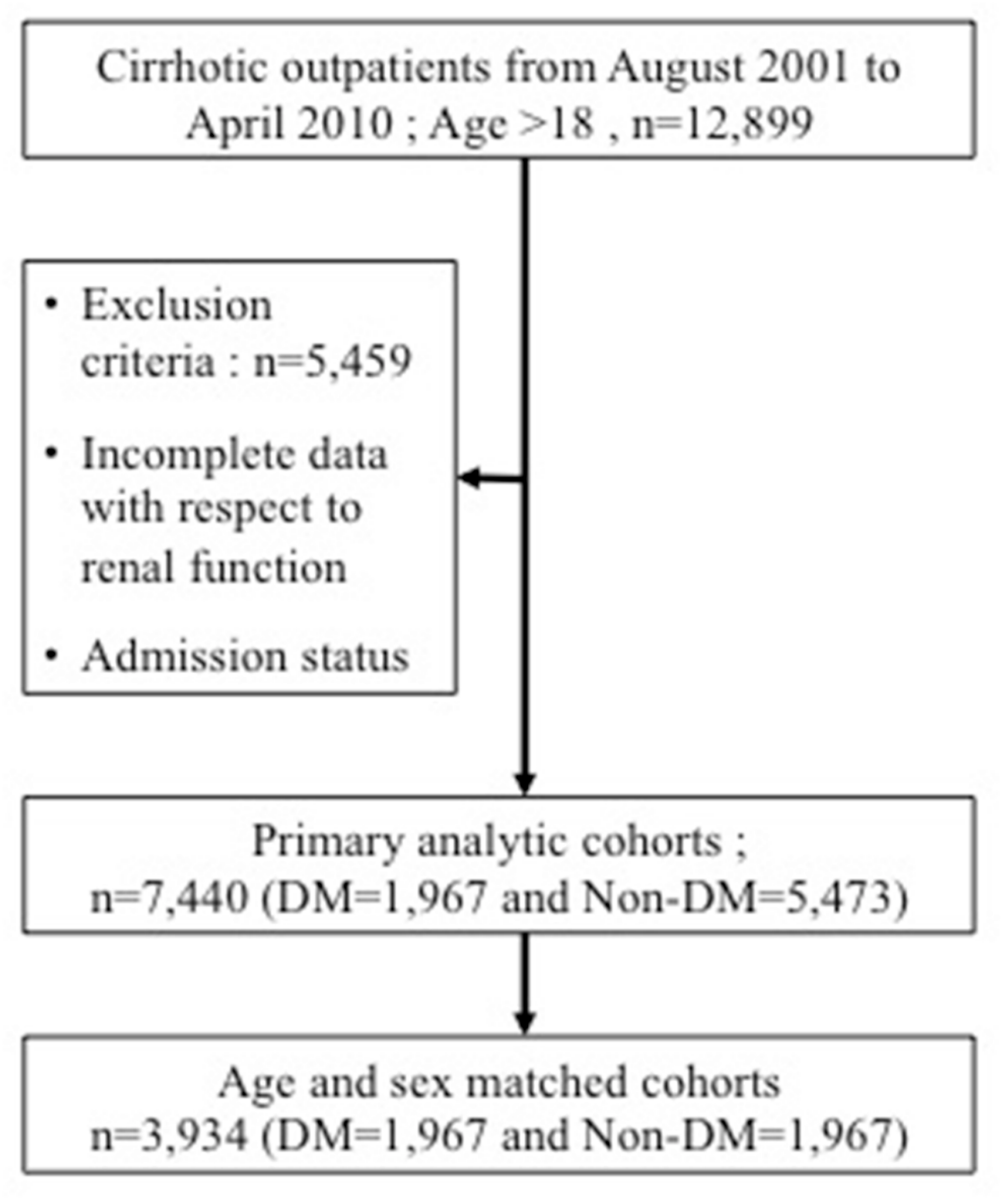
Flowchart of selection of study population

Among the enrolled cohort, we used case control matching with age difference within three years, sex and presence of diabetics or not. Totally, we obtained 1,967 matched pairs (Figure 3). The severity of liver disease was calculated from Child-Turcott-Pugh (CTP) and MELD scores. Data on the etiology of the liver disease including viral and alcoholic causes were historically and serologically collected. The etiology of cirrhosis was defined to be chronic hepatitis B virus infection with long-standing (>6 months) HBsAg positivity; chronic hepatitis C virus (HCV) infection with detectable both antibodies against HCV (anti-HCV) and serum HCV-RNA. All patients who were classified as having alcoholic cirrhosis had ingested >80 g daily of alcohol for a decade or more.

Based on the admission data, each patient had the CTP score (range: 5-15) and Child class calculated according to the suggestion by Pugh et al. [44], while the MELD score (range: 6-40) was calculated according to the formula proposed by Kamath et al. [2], which was a slight modification of the risk score used in the original TIPS model [45].

### Estimation of GFR

Serum creatinine (SCr) was measured by the isotope dilution mass spectrometry (IDMS)-traceable enzymatic method in a Roche Cobas Integra 400 at the Mackay Memorial Hospital. GFR was estimated using by three different equations including the 4-and 6-variable MDRD Study [46, 47], the Chronic Kidney Disease Epidemiology Collaboration creatinine (2009) CKD-EPI [6]equation.

We calculated eGFR using the IDMS-traceable 4-variable MDRD Study equation [46]: eGFR MDRD :175 × Scr^1.154^ × age^-0.203^ × (0.742 if female), 6 variable MDRD = 170 × Scr^-0.999^ × age^-0.176^ × (0.762 if patient is female) × (BUN)^-0.170^ × (Albumin)^0.318^ and also using the CKD-EPI equation: 141 × min (SCr/k, 1)^α^ × max (SCr/k, 1)^-1.209^ × 0.993^Age^ × 1.018 [if female], where k is 0.7 for female and 0.9 for male, α is -0.329 for female and -0.411 for male, min indicates the minimum of SCr/k or 1, and max indicates the maximum of SCr/k or 1.

### Definition

CKD was defined by either a low eGFR (<60 ml/min per 1.73 m^2^) or the presence of albuminuria based on spot urine samples using sex-specific cut offs [48]. CKD stages were defined according to clinical practice guidelines developed under the National Kidney Foundation's Kidney Disease Outcomes Quality Initiative (KDOQI). Diabetes was defined as a history of diabetes (self-report or retinopathy), fasting blood glucose level >126 mg/dL, or nonfasting blood glucose level >200 mg/dL in the absence of self-report or medication use, use of medications to treat diabetes. CVD history was defined as self-reported history of heart attack, heart angioplasty, heart failure, bypass surgery, abnormal heart rhythm, or stroke. Dyslipidemia was defined as total cholesterol level >200 mg/dL or triglyceride level >150 mg/dL. Obesity, defined as BMI >30 kg/m^2^. Proteinuria defined as a dipstick urinalysis score of 1+ or greater (equivalent to≥30 mg/dL) or Urine rotein-to-creatinine ratio ≥ 150 mg/mg or urine albumin-to-creatinine ratio (ACR) ≥30 mg/g.

### Statistical analysis

To test differences in characteristics between participants with and without diabetes, we used mean and standard deviation for normally distributed [median and interquartile range for non-normally distributed] continuous variables, N (%) for categorical variables. The Mann-Whitney U test and chi-square test were used because of the existence of outliers, high variability, and skewed distributions. One-way analysis of variance was used to test differences in continuous variables between participants of different groups. We used logistic regression, expressed as odds ratio (OR) and 95% confidence interval (CI), to describe the association of CKD stages and other clinical characteristics with diabetes (dependent variable). Separate models were constructed using MDRD-4, MDRD-6 and CKD-EPI for eGFR and adjusted model for age, sex, dyslipidemia, CVD, hemoglobin, albumin, and uric acid.

We performed conditional logistic regression for matched group, expressed as odds ratio (OR) and 95% confidence interval (CI), to describe the association of CKD stage 1, stage 2 and CKD stage 3~5 with diabetes compared to non-CKD group. Adjusted model 1 used the MDRD-4 equation to define CKD; adjusted model 2 used the MDRD-6 equation; adjusted model 3 used the CKD-EPI equation. We used the conventional p<0.05 for statistical significance in this study. All statistical analyses were performed using SPSS software (version 17.0, SPSS Inc., Chicago, IL, USA).
